# Shape-Controlled Syntheses of Magnetite Microparticles and Their Magnetorheology

**DOI:** 10.3390/ijms20153617

**Published:** 2019-07-24

**Authors:** Hiroya Abe, Takashi Naka, Kazuyoshi Sato, Yoshikazu Suzuki, Masami Nakano

**Affiliations:** 1 Joining and Welding Research Institute, Osaka University, Osaka 567-0047, Japan; 2 National Institute for Materials Science, Ibaraki 305-0047, Japan; 3 Graduate School of Science & Technology, Gunma University, Gunma 376-8515, Japan; 4 Faculty of Pure and Applied Sciences, University of Tsukuba, Ibaraki 305-8573, Japan; 5 New Industry Creation Hatchery Center, Tohoku University, Sendai 980-8577, Japan

**Keywords:** magnetite, shape-controlled, octahedral-shape, microparticle, magnetorheology

## Abstract

Magnetic microspheres in a concentrated suspension can be self-assembled to form chain structures under a magnetic field, resulting in an enhanced viscosity and elasticity of the suspension (i.e., the magnetorheological (MR) effect). Recently, interest has been raised about the relationship between nonspherical particles, such as octahedral particles and the MR effect. However, experimental studies have not made much progress toward clarifying this issue due to the difficulty associated with synthesizing microparticles with well-defined shapes and sizes. Here, we presented a method for the shape-controlled synthesis of magnetite (Fe_3_O_4_) microparticles and investigated the MR effects of two suspensions prepared from the two shape-controlled samples of Fe_3_O_4_ microparticles. Our method, which was based on the polyol method, enabled the preparation of spherical and octahedral Fe_3_O_4_ microparticles with similar sizes and magnetic properties, through a reduction of α-FeOOH in a mixed solvent of ethylene glycol (a polyol) and water. The water played an important role in both the phase transition (α-FeOOH to Fe_3_O_4_) and the shape control. No substantial difference in the MR effect was observed between an octahedral-particle-based suspension and a spherical-particle-based one. Therefore, in this study, the shape of the microparticles did not strongly influence the MR effect, i.e., the properties of the chain structures.

## 1. Introduction

A magnetorheological (MR) fluid is a magneto-responsive soft material that undergoes a reversible transition from a liquid-like to a near-solid state under the influence of an external magnetic field (i.e., the MR effect) [1]. This property makes MR fluids good candidates for applications in mechanical systems such as passive MR dampers [2], haptic interfaces [3], and human-friendly robots [4]. This material is a two-phase system consisting of magnetic particles and a carrier liquid [1]. The mechanism responsible for the MR effect is an attractive interaction between the induced magnetic dipoles, which causes the suspended particles to form chain structures aligned parallel to an applied magnetic field. Such chain structures can substantially increase the viscosity and yield stress of an MR fluid on a millisecond timescale.

Micron-sized iron particles with a spherical shape are used as MR fluids [1,5,6]. If the particles are nano-sized, Brownian motion prevents the formation of chain structures [7,8]. In fact, ferrofluids, which are colloidal dispersions of magnetic nanoparticles approximately 10 nm in diameter, exhibit a weak MR effect because of predominant Brownian motion [8]. As another material for MR fluids, magnetite (Fe_3_O_4_) particles have been studied because they exhibit better chemical stability against oxidation and are less prone to sedimentation than Fe particles [9,10,11].

Over the past few decades, much effort has been devoted to synthesizing nanoscale particles with various shapes such as cubic, tetrahedral, and octahedral [12,13,14]. Consequently, questions have arisen about the relationship between nonspherical particles and the MR effect [15]. Jung et al. synthesized octahedral-shaped Fe_3_O_4_ nanoparticles [16] and expected them to form stronger chain structures than spherical particles due to interparticle friction. This hypothesis was based on the presumption that chains of octahedral-shaped particles would have a larger contact surface than chains of spherical particles. Expecting the same mechanism, Han et al. synthesized triangular-shaped Fe_3_O_4_ nanoparticles [10]. Although MR effects were observed in the two aforementioned fluids, it is unclear if such nanoscale surface contact enhances the MR effects. The use of micron-sized octahedral particles in such investigations might be preferable because they are negligibly influenced by Brownian motion under magnetic fields. However, the development of a simple and reliable method for synthesizing magnetic microparticles with well-defined shapes and sizes remains a challenge.

In this study, we developed a facile polyol-based method for synthesizing shape-controlled Fe_3_O_4_ microparticles. The polyol method can produce microparticles of metals and metal oxides via the reduction of precursors in a polyol solvent (typically ethylene glycol (EG)) [17]. Our modified polyol method enabled the preparation of spherical and octahedral Fe_3_O_4_ microparticles with similar sizes and magnetic properties through reduction of α-FeOOH (solid precursor) in a mixed solvent of EG and water. In this method, water plays important roles in both phase transition (α-FeOOH to Fe_3_O_4_) and shape control. Two suspensions were prepared from the spherical and octahedral microparticles, and their MR effects were compared to directly probe the effect of particle shape on the MR effect.

## 2. Results

### 2.1. Syntheses of Spherical and Octahedral Fe_3_O_4_ Microparticles

In this study, Fe_3_O_4_ microparticles were prepared via reduction of α-FeOOH in a solvent mixture of water–EG at 200 °C. Moreover, their shapes were controlled by varying 9 vol. % and 12 vol. % water concentrations of the mixed solvents.

**Figure 1:**
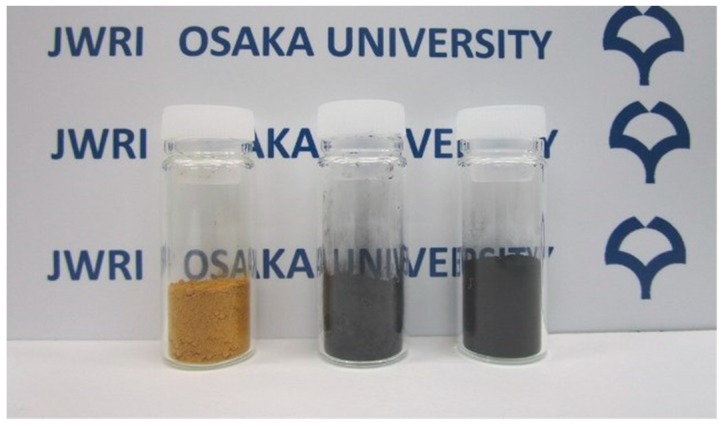
Photographs of (**left**) α-FeOOH powder used as a precursor; (**center**) the product prepared by reducing α-FeOOH in the mixed solvent with 9 vol. % water concentration at 200 °C for 24 h; (**right**) the product prepared by reducing α-FeOOH with 12 vol. % water concentration at 200 °C for 48 h.

**Figure 2:**
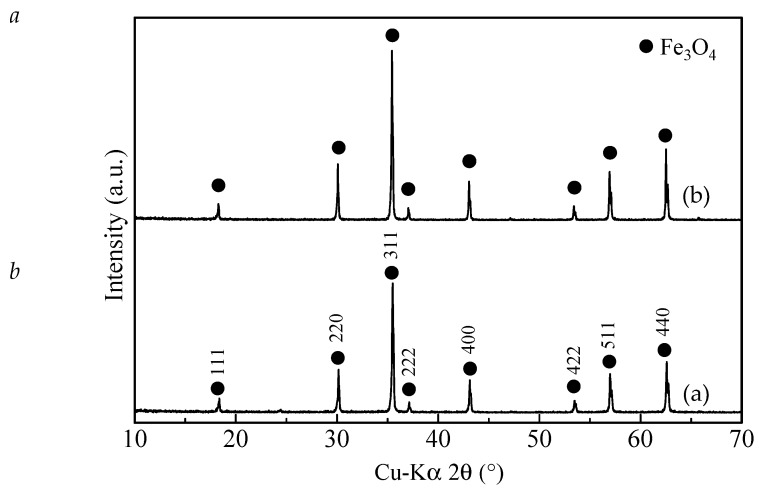
X-ray diffraction patterns of (**a**) the powder prepared by the reduction of α-FeOOH in the mixed solvent with 9 vol. % water concentration at 200 °C for 24 h; (**b**) the powder prepared by reduction of α-FeOOH with 12 vol. % water concentration at 200 °C for 48 h.

The phase transformation of α-FeOOH to Fe_3_O_4_ was accompanied by a color change (yellow to black) of the powder during reductive aging of α-FeOOH in both mixed solvents (Figure 1 and Figure 2). Yellow and black are typical colors of α-FeOOH and Fe_3_O_4_, respectively [18]. The X-ray diffraction (XRD) patterns of the two black powders demonstrate their crystalline nature, and the peaks match well with the standard reflections for Fe_3_O_4_ (#019-0629, PDF-2 database, ICDD, Newtown Square, PA, USA). The peaks are sharp and intense, indicating good crystallinity of the samples. Almost no characteristic peaks arising from α-FeOOH (Figure S1) or other iron oxides or oxyhydroxides were observed. The aging times for 9 vol. % and 12 vol. % water concentrations were 24 h and 48 h, respectively, although the exact aging times needed for the two water concentrations have not yet been examined. The black powder (Fe_3_O_4_) was not obtained when aging the yellow powder (α-FeOOH) in only the EG solvent (in the absence of water) even for 72 h at 200 °C.

**Figure 3:**
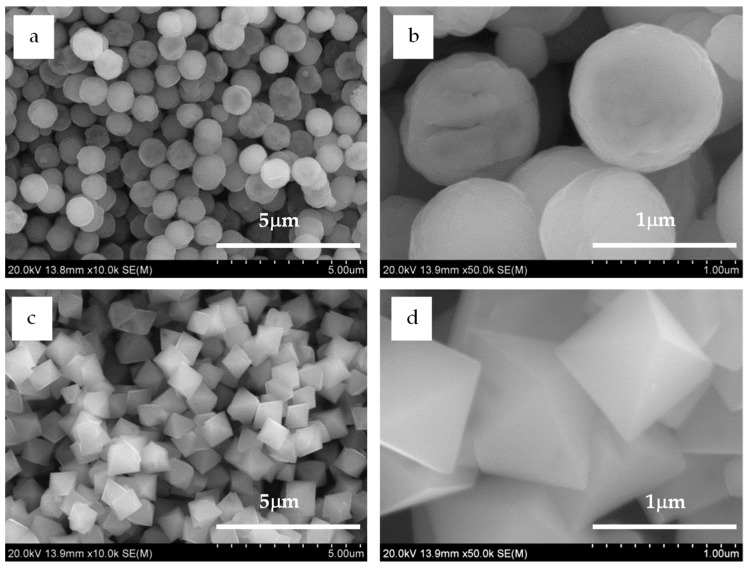
Typical scanning electron microscopy images of the products (**a**,**b**). Spherical Fe_3_O_4_ particles prepared by reduction of α-FeOOH in the mixed solvent with 9 vol. % water concentration at 200 °C for 24 h; (**c**,**d**) octahedral Fe_3_O_4_ particles prepared by reduction of α-FeOOH with 12 vol. % water concentration at 200 °C for 48 h.

The α-FeOOH particles were needle-shaped (Figure S2). They were transformed mostly to spherical and octahedral Fe_3_O_4_ microparticles via aging in EG containing 9 vol. % and 12 vol. % water concentration, respectively, as shown in Figure 3. The spherical microparticles featured a relatively narrow size distribution, and their average diameter was 1.00 ± 0.13 µm (Figure S3a). Their surface images reflected the polycrystalline nature (Figure 3b), indicating that each spherical microparticle comprised small primary grains. The aging time was another important parameter to control the spherical shape because with an increase in aging time, the Fe_3_O_4_ microparticles did not maintain the spherical shape.

For the octahedral microparticles, the longest dimension of each particle was measured as it appeared in the scanning electron microscopy images, following the measuring procedure of Vereda et al. [19]. A narrow size distribution was also observed for the octahedral microparticles (Figure S3b), where the average diameter was 1.10 ± 0.15 µm—slightly larger than that of the spherical microparticles. The Fe_3_O_4_ microparticles maintained the octahedral shape with increasing aging time. The octahedral shape was the main theoretical morphology [20]; therefore, their shape suggested that each particle was a single crystal.

**Figure 4:**
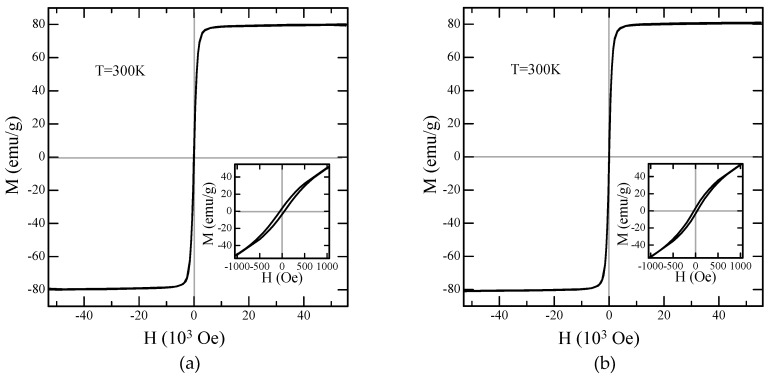
(**a**) Mass magnetization *M* as a function of the applied external magnetic field H measured for the spherical Fe_3_O_4_ microparticles at 300 K. (**b**) Mass magnetization for the octahedral Fe_3_O_4_ microparticles.

To characterize the magnetic properties of the synthesized particles, we recorded their magnetization curves at room temperature (300 K). The curves were almost the same for both the octahedral and spherical Fe_3_O_4_ microparticles, as shown in Figure 4. The curves exhibited very small hysteresis loops that were barely observable at the scale shown in Figure 4a,b. However, they were apparent in the magnified image (inset). The saturation magnetizations of the Fe_3_O_4_ microparticles with spherical and octahedral shapes were 80.2 emu/g and 81.0 emu/g, respectively, and their coercivities were 47 Oe and 36 Oe, respectively.

Thus, the modified polyol process presented here is a straightforward and effective synthesis strategy for preparing spherical and octahedral Fe_3_O_4_ microparticles with similar sizes and magnetic properties. Due to these features, the resulting two shape-controlled microparticles might be an ideal system to directly probe the shape dependency of magnetorheology.

### 2.2. Magnetorheology of The Shape-Controlled Fe_3_O_4_ Microparticles

**Figure 5:**
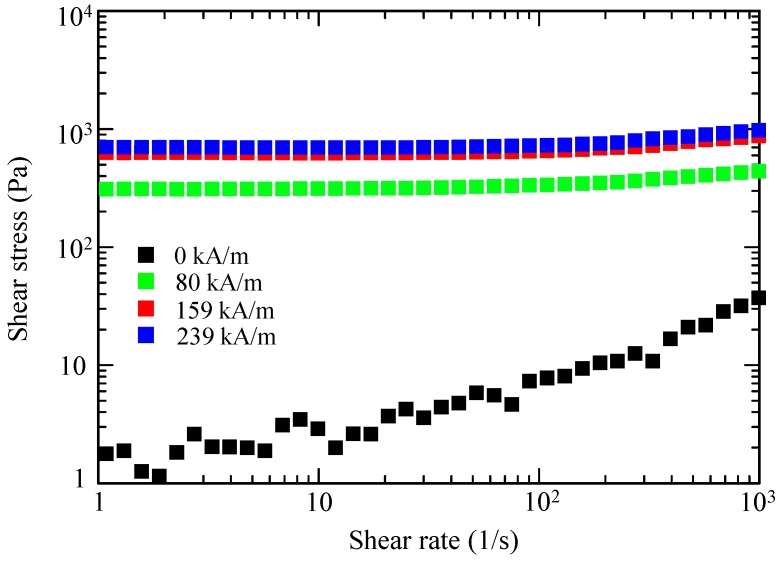
Flow curves under the magnetic fields for a Fe_3_O_4_ suspension (10 vol. %) containing spherical particles.

**Figure 6:**
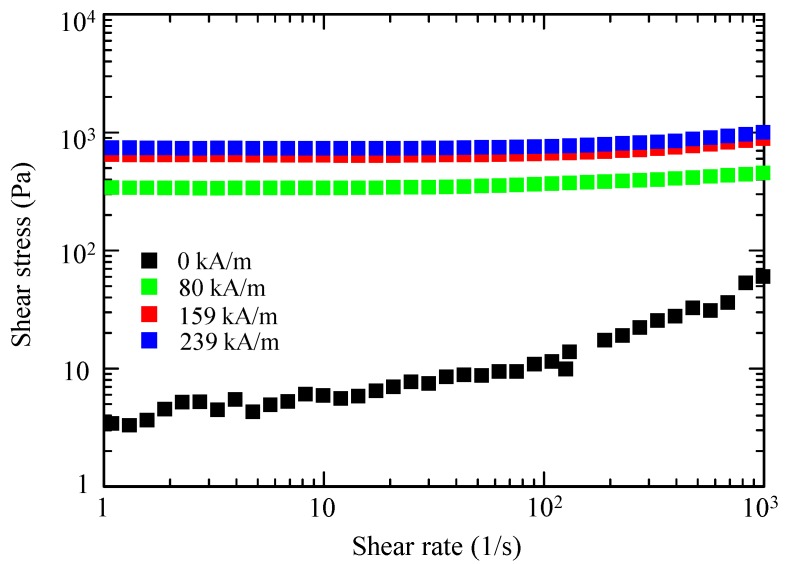
Flow curves under the magnetic fields for a Fe_3_O_4_ suspension (10 vol. %) containing octahedral particles.

Suspensions of both the spherical and octahedral Fe_3_O_4_ microparticles were prepared. EG was used as a carrier liquid herein. Figure 5 and Figure 6 show the flow curves of the two suspensions with a solid fraction of 10 vol. %, using a controlled shear-rate test under a range of magnetic flux densities, plotted on a log–log scale. The flow curve provides information about the dependence of the shear stress on the shear rate.

At zero magnetic fields, both fluids exhibited Newtonian-like behavior, where the shear stress increased linearly with increasing shear rate. The shear stress of the octahedral-particle-based MR fluid was slightly higher than that of the spherical-particle-based MR fluid, likely because of the stronger deflection of the flow lines of the fluid flowing around the octahedral particles and the stronger particle–particle interaction among the octahedral particles.

When subjected to a magnetic field, both suspensions showed an increase of their shear stress with increasing magnitude of the magnetic field. Notably, no substantial difference existed in the MR effect between the spherical-based and octahedral-based suspensions. These characteristics were distinctive solid-like behaviors with a clear yield stress. The observed solid-like or plastic behavior can usually be characterized by the Bingham fluid model [5,16,21], which can be explained by the formation of chain-like structures or magnetic particle columns in a fluid [1,5].

**Figure 7:**
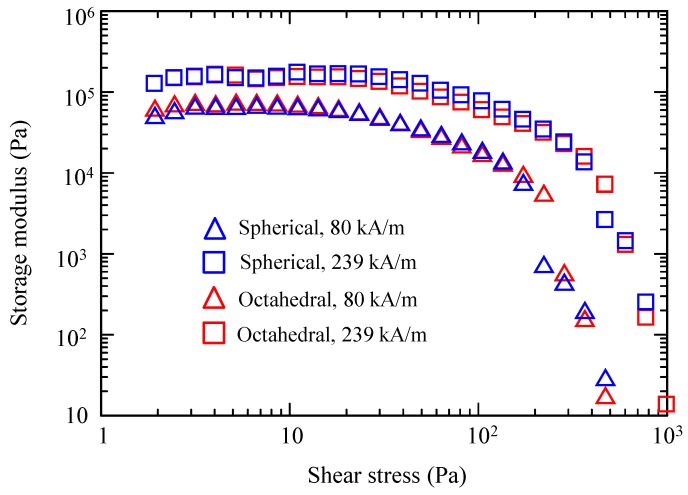
Storage modulus as a function of the shear stress amplitude, for the spherical-based and the octahedral-based suspensions.

In addition, oscillatory shear measurements were performed to further explore the shape dependence. Figure 7 shows the storage moduli (G’) of both suspensions as a function of the shear stress amplitude at different magnetic fields (80 and 239 kA/m). The G’ values of the suspensions did not differ significantly. In each suspension, G’ increased as the strength of the magnetic field increased. Pseudoplateaus were observed at low shear stress values, which are referred to as linear viscoelastic regions. G’ values in this region provide information about the strength of the field-induced chain structures [22]. As the linear viscoelastic G’ values of the microparticle suspensions were very similar, the strengths of the field-induced spherical and octahedral microparticle structures would be nearly identical. Therefore, the particle shape did not substantially influence the magnetorheology in our comparative studies.

## 3. Discussion

### 3.1. Synthesis Mechanism

**Figure 8:**
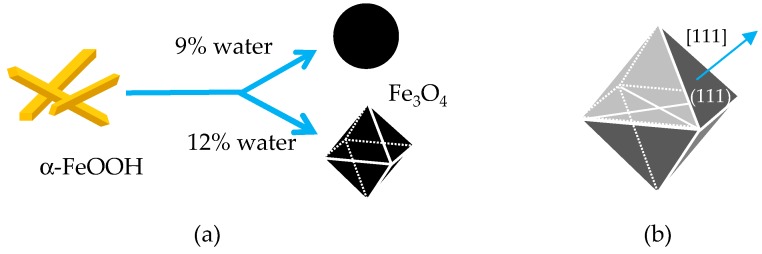
(**a**) Schematic of Fe_3_O_4_ with disparate shapes resulting from different water concentrations in the EG–water mixed solvent used in the synthesis; (**b**) Fe_3_O_4_ octahedron of a single crystal enclosed by eight {111} planes. <111>: easy axis of magnetization.

Figure 8a depicts a simple schematic of the two shape-controlled syntheses of Fe_3_O_4_ microparticles. The two main features in the syntheses were (1) the phase transformation of α-FeOOH to Fe_3_O_4_ under EG-based reductive aging in the presence of water and (2) the two shape-controls that depend on the water concentration. We, therefore, discuss these two points in this order.

#### 3.1.1. Phase Transformation

In the polyol method, particle formation proceeded via solution—specifically, dissolution of the solid precursor, reduction of metal ions to a lower valence state, and precipitation of the resultant solid phase [17]. In this study, the phase transition did not occur in the absence of water. Therefore, the water functioned as a dissolving agent for α-FeOOH. Once the Fe^3+^ ions were released by dissolution into the solution, EG (C_2_H_6_O_2_) partially reduced the Fe^3+^ ions to Fe^2+^ ions. Subsequently, Fe_3_O_4_ was precipitated through hydrolysis of a mixture of Fe^3+^ and Fe^2+^ ions. According to the literature [17], the main oxidation product of EG in the polyol process is diacetyl (C_4_H_6_O_2_). Thus, the phase transformation of α-FeOOH to Fe_3_O_4_ could be described by the following reactions:

(1) $$\mathrm{FeOOH}+H_{2}O\to {\mathrm{Fe}}^{3+}+3{\mathrm{OH}}^{-}$$

(2) $$2\left(C_{2}H_{6}O_{2}\right)\to 2e^{-}+C_{4}H_{6}O_{2}+2H_{2}O+2H^{+}$$

(3) $$6\mathrm{FeOOH}+6H_{2}O+2\left(C_{2}H_{6}O_{2}\right)\to 2{\mathrm{Fe}}_{3}O_{4}+C_{4}H_{6}O_{2}+12H_{2}O$$

As Fe_3_O_4_ is a more thermodynamically stable phase than = α-FeOOH, in a reducing environment [23], it could be considered that reactions (1)–(3) proceeded spontaneously.

#### 3.1.2. Shape Control

Inorganic particles often assume their thermodynamically favored shapes during slow crystal growth [24]. For Fe_3_O_4_, the thermodynamically favored shape is octahedral [19,20]. Fe_3_O_4_ has an inverse spinel structure with oxygen forming a face-centered cubic (FCC) closed packing. In such FCC crystals, the surface energies for the FCC facets scale accordingly: γ(111) < γ(100) < γ(101), which means that the crystals usually exist as an octahedron surrounded by {111} facets (Figure 8b). In our experiments, the octahedral particles were obtained after the aging process in an EG–water solvent with 12 vol. % water, indicating that this condition would result in a sufficiently slow crystal growth to enable the growth of the octahedral particles.

α-FeOOH is very poorly soluble (solubility product *K*_sp_, log*K*_sp_ <−41 at 25 °C [25]). In addition, EG is diluted by water, i.e., the concentration of the reductant (EG) is lowered. Therefore, the monomers, which are the minimum subunits of bulk Fe_3_O_4_, are considered to be supplied considerably slowly into the reaction system and to be taken up by the preformed nuclei.

The supply rate of the monomers in the presence of 9 vol. % water would also be slow. However, it must be faster than that in 12 vol. % water because the reductant is less diluted. In fact, the aging time with 9 vol. % water was shorter than that with 12 vol. % water. Thermodynamically unstable structures are often obtained at faster growth rates [24,26]. In our case, spherical particles with a polycrystalline structure were obtained, which were similar to those synthesized by EG-based reductive aging of FeCl_3_·6H_2_O in the presence of sodium acetate and a surfactant [27,28] and those prepared by the oxidative aging of Fe(OH)_2_ in the presence of KNO_3_ [29].

The observed nearly monodispersed characteristics for both particle types suggest that a burst of nucleation occurs in our synthesis system. The burst of nucleation enables the separation of homogeneous nucleation and growth processes, which is known to be an effective strategy for preparing monodispersed particles [30]. The sufficiently slow supply of monomer as a result of the dissolution of a solid precursor has been demonstrated to result in a burst of nucleation for monodispersed microparticles of iron oxides [29,31,32]. In our study, the slow supply could be achieved by the reductive hydrolysis of α-FeOOH in solution; notably, changing the supply rate could enable shape control in the absence of a surfactant. The elucidation of the detailed mechanism remains in progress and will be reported in a future study.

To the best of our knowledge, the system reported here is the simplest synthetic system reported for producing Fe_3_O_4_ microparticles. It is attractive because the precursor (α-FeOOH) is inexpensive and the procedure itself is economical because it does not involve additives such as a base for hydrolysis or surfactants for shape control, which enables an easy scale-up. Due to the simplicity of this system, we synthesized sufficient amounts of Fe_3_O_4_ microparticles to prepare MR fluids.

### 3.2. Shape Effect on Magnetorheology

Recently, octahedral particles have been speculated to enhance the MR effect [16,22]. Such magnetic particles would make stronger particle chains than spherical magnetic particles because the chains of the octahedral particles have a larger contact surface [16]. In this system, a remarkable contribution of the interparticle friction is expected. However, the results obtained in the present study indicated that the effect of particle shape appears to be less important.

The Fe_3_O_4_ chosen as a magnetic material herein exhibited magnetocrystalline anisotropy—the <111>, <110>, and <100> crystallographic directions corresponded to the easy, intermediate, and hard axes of magnetization, respectively [14]. An octahedral Fe_3_O_4_ particle is enclosed by eight {111} planes, which are perpendicular to the <111> direction (Figure 8b). Therefore, when subjected to a magnetic field, octahedral Fe_3_O_4_ particles would form chains along the easy axis by connecting {111} faces, leading to a larger contact surface compared with that of spherical particles. In fact, such face-to-face assembled structures have been observed for octahedral Fe_3_O_4_ nanoparticles under a magnetic field [13,14]. However, the MR effect of the octahedral-based and spherical-based suspensions was detected to be almost similar, as shown in Figure 5, Figure 6 and Figure 7.

The results obtained in this study suggest that a mechanism might exist that made negligible contribution of the interparticle friction in our octahedral-based suspension. This mechanism might be related to particle concentration. Vereda et al. have reported that a 1 vol. % suspension containing faceted particles (predominantly octahedral particles) enhanced the MR effect compared to their spherical-based suspension; however, this MR enhancement decreased as the particle concentration increased to 5 vol. % [22]. Our particle concentration was 10 vol. % and almost no enhancements were observed, as mentioned above. We, thus, speculate that the interaction among the magnetic field-aggregated particles, rather than the interparticle friction of the octahedral microparticles, might predominantly cause the viscosity or elasticity to increase at particle concentrations exceeding 10 vol. %.

## 4. Materials and Methods

### 4.1. Shape-Controlled Synthesis of Fe_3_O_4_ Microparticles

All chemicals were of analytical grade and were used as received without further purification. α-FeOOH powder (4.26 g/cm^3^) was purchased from Kojundo Chemical Laboratory Co., Ltd. (Saitama, Japan). EG was purchased from Kishida Chemical Co., Ltd. (1.11 g/cm^3^, Osaka, Japan). Water used in all syntheses was distilled and deionized.

Fe_3_O_4_ microparticles were synthesized via reductive aging of α-FeOOH in a mixed solvent of EG and water. The particle shape was controlled by adjusting the water concentration. In a typical procedure for spherical-shaped particles, 15 g of α-FeOOH was placed in a Teflon-lined stainless steel autoclave (San-ai Kagaku Co., Ltd., Nagoya, Japan) with a capacity of 50 mL. The autoclave was then filled with 48 g of the EG–water mixed solvent (water concentration—9 vol. %). After the autoclave was sealed, it was heated at 200 °C for 24 h. The resultant Fe_3_O_4_ microparticles were repeatedly washed with water and ethanol and dried in a vacuum oven at 50 °C for 24 h. After drying, the weights of the microparticles were measured to calculate the yield. The octahedral-shaped particles were prepared by the same procedure except for the water content (12 vol. %) and the aging time (48 h). The yield of these syntheses was ca. 86%. The loss was mainly due to the microparticles lost during the washing.

### 4.2. Preparation of Fe_3_O_4_ Microparticle-Based Suspensions

EG was used as a carrier liquid. Spherical and octahedral Fe_3_O_4_ microparticles were dispersed in the EG via shaking and ultrasonication to prepare two different suspensions. The concentration of the microparticles in both fluids was 10 vol. %.

### 4.3. Characterization of Fe_3_O_4_ Microparticles and Evaluation of MR Fluids

The morphologies of the as-synthesized samples were observed by field-emission scanning electron microscopy (FE-SEM, SU-70, Hitachi High-Tech). Their phase structures were analyzed by XRD (Multiflex, Cu-Kα, 40 kV and 40 mA, Rigaku, Tokyo, Japan). DC magnetization was measured at room temperature with a conventional superconducting quantum interference device magnetometer (MPMS-XL, Quantum Design, San Diego, CA, USA). Steady MR responses of the Fe_3_O_4_ microparticle-based suspensions were measured at room temperature, using a magnetic-field-applicable parallel-disk sensor with an electromagnetic system (MR-101N, EKO Instruments, Tokyo, Japan) attached to a high-precision rheometer (RheoStress RS-150, HAAKE, Germany). A parallel-plate fixture with a diameter of 20 mm was used, and the gap was set to 300 µm. A magnetic field was applied perpendicular to the shear flow direction. Current control regulated the generated magnetic field from 0 to 239 kA/m. The samples stood for a few seconds after application of the magnetic fields, and then the steady-state and oscillatory shear measurements were performed at room temperature. In steady-state measurement, the samples were sheared at shear rates varying from 1 to 1,000 s^−1^ and the corresponding shear stresses were recorded. In the oscillatory shear measurement, an oscillatory shear stress was applied with an amplitude ranging between 10^−^^1^ and 10^3^ Pa at a constant frequency of 1 Hz.

## Supplementary Materials

Supplementary materials can be found at https://www.mdpi.com/1422-0067/20/15/3617/s1.
