# Supplementary material for: Shape-Controlled Syntheses of Magnetite Microparticles and Their Magnetorheology
Source: Int J Mol Sci. 2019 Jul 24;20(15):3617. doi: 10.3390/ijms20153617 (PMC6695728; doi:10.3390/ijms20153617)
Supplement: Supplementary file 1 [file ijms-20-03617-s001.pdf]

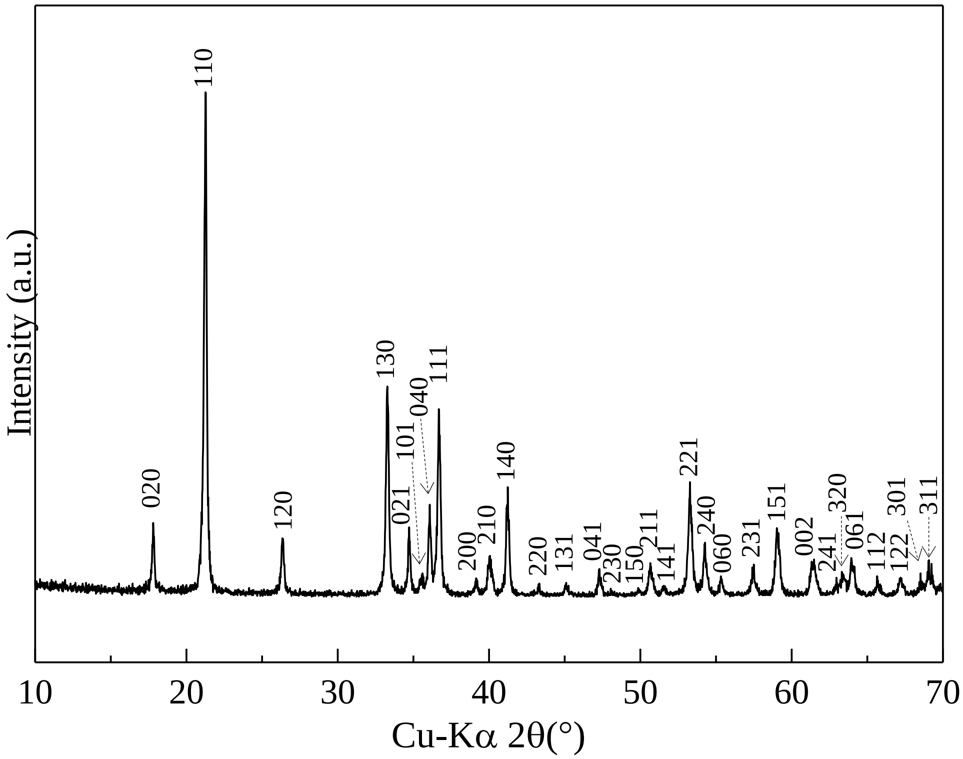

Figure S1. X-ray diffraction patterns of α-FeOOH. All peaks are well-indexed with α-FeOOH ICDD PDF #01-081-0462.

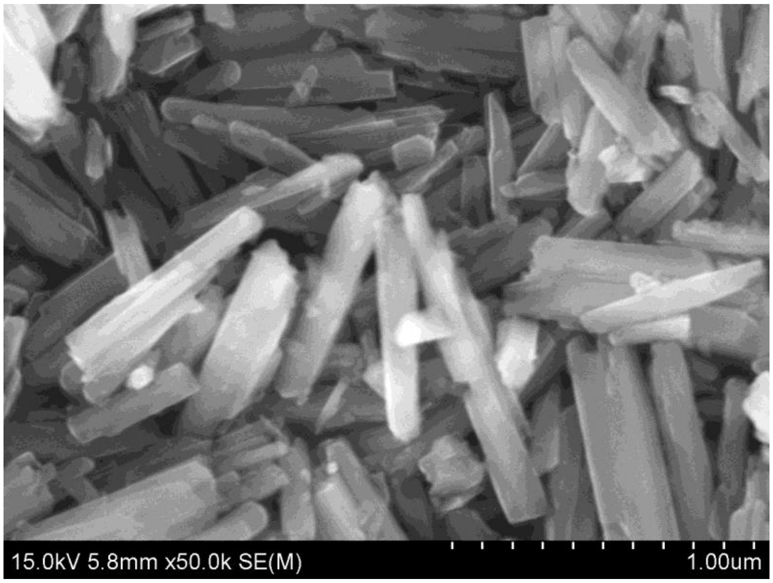

Figure S2. An SEM image of α-FeOOH

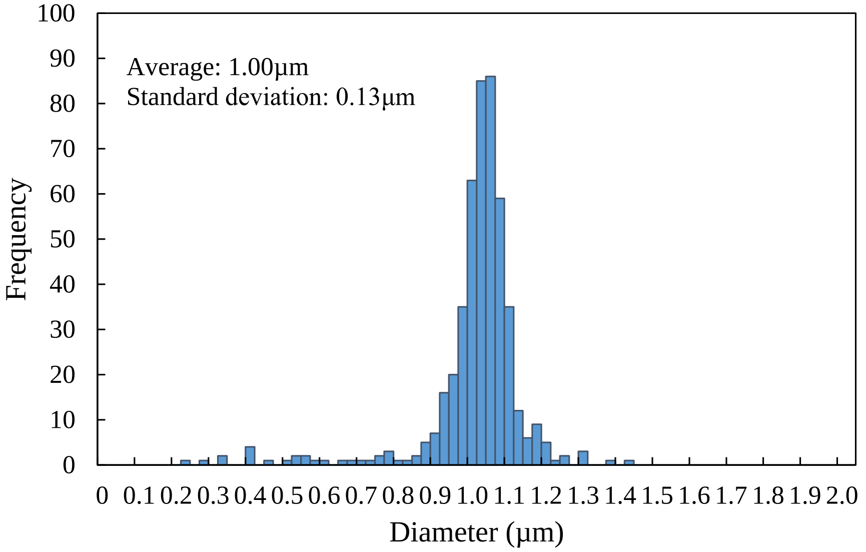

(a)

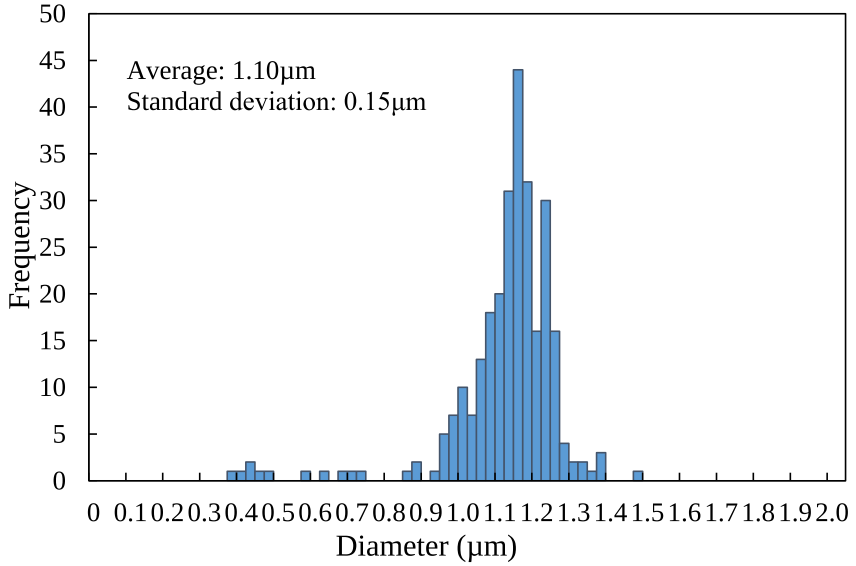

(b)

Figure S3. Size distributions of (**a**) spherical Fe_3O_4 microparticles (480 particles); (**b**) octahedral Fe_3O_4 microparticles (particles 277 particles)
